# Global profiling of histone and DNA methylation reveals epigenetic-based regulation of gene expression during epithelial to mesenchymal transition in prostate cells

**DOI:** 10.1186/1471-2164-11-669

**Published:** 2010-11-25

**Authors:** Xi-Song Ke, Yi Qu, Yang Cheng, Wen-Cheng Li, Varda Rotter, Anne Margrete Øyan, Karl-Henning Kalland

**Affiliations:** 1 The Gade Institute, University of Bergen, Laboratory Building, 5. etg. Vest, Helse Bergen, NO-5021 Bergen, Norway; 2 Fritz-Frey-Str. 6,69121, Heidelberg, Germany; 3 Urological Department, Union Hospital, Tongji Medical College, Huazhong University of Science and Technology, Wuhan 430022, PR China; 4 Department of Molecular Cell Biology, Weizmann Institute of Science, Rehovot, Israel; 5 Department of Microbiology, Haukeland University Hospital, Bergen, Norway

## Abstract

**Background:**

Previously we reported extensive gene expression reprogramming during epithelial to mesenchymal transition (EMT) of primary prostate cells. Here we investigated the hypothesis that specific histone and DNA methylations are involved in coordination of gene expression during EMT.

**Results:**

Genome-wide profiling of histone methylations (H3K4me3 and H3K27me3) and DNA methylation (DNAMe) was applied to three cell lines at different stages of a stepwise prostate cell model involving EMT and subsequent accumulation of malignant features. Integrated analyses of epigenetic promoter modifications and gene expression changes revealed strong correlations between the dynamic changes of histone methylations and gene expression. DNA methylation was weaker associated with global gene repression, but strongly correlated to gene silencing when genes co-modified by H3K4me3 were excluded. For genes labeled with multiple epigenetic marks in their promoters, the level of transcription was associated with the net signal intensity of the activating mark H3K4me3 minus the repressive marks H3K27me3 or DNAMe, indicating that the effect on gene expression of bivalent marks (H3K4/K27me3 or H3K4me3/DNAMe) depends on relative modification intensities. Sets of genes, including epithelial cell junction and EMT associated fibroblast growth factor receptor genes, showed corresponding changes concerning epigenetic modifications and gene expression during EMT.

**Conclusions:**

This work presents the first blueprint of epigenetic modifications in an epithelial cell line and the progeny that underwent EMT and shows that specific histone methylations are extensively involved in gene expression reprogramming during EMT and subsequent accumulation of malignant features. The observation that transcription activity of bivalently marked genes depends on the relative labeling intensity of individual marks provides a new view of quantitative regulation of epigenetic modification.

## Background

Carcinomas arise from normal epithelial tissues in a multistep process. The breakdown of epithelial cell homeostasis leading to aggressive cancer progression corresponds with the loss of epithelial characteristics and the acquisition of migratory phenotypes, referred to as epithelial to mesenchymal transition (EMT), and is believed to be a crucial event in tumor progression and endows cancer cells with invasive and metastatic competence [1-3]. In a transformation attempt, however, we have observed complete EMT from benign prostate epithelial cells (EP156T) to cells with a mesenchymal phenotype (EPT1) without malignant transformation [4]. To achieve transformed prostate cells, EPT1 cells were kept growing in extended saturation density culture to select for cells overriding quiescence. Many foci formed in EPT1 cell monolayers. Cells (EPT2) were isolated from the foci and were found to have acquired several malignant features, such as anchorage independent growth, much higher abilities to proliferation at confluence, increased resistance to apoptosis and much lower dependence on external growth factors compared with EP156T and EPT1 cells. Both cytogenetic and DNA fingerprinting analyses revealed genetic identity of the three cell lines and confirmed progeny authenticity of the cell model. EPT2 cells did not, however, form tumors in animals suggesting their being at an early transformation stage and additional induction is required for full malignant transformation [5]. This stepwise cell model provides a good opportunity to understand the mechanisms of EMT and its role in subsequent accumulation of malignant features *in vitro*.

Epigenetic modifications, especially histone and DNA methylations, have a large impact on the regulation of gene expression and are critical in establishing patterns of gene repression during development [6]. Previous genome-wide maps of histone H3 lysine 4 and lysine 27 tri-methylation (H3K4me3 and H3K27me3) showed a very clear correlation between H3K4me3 and expressed genes and H3K27me3 and repressed genes in embryonic stem cells [7-10], T cells [11], hematopoietic stem cells/progenitor cells [12] and in prostate cancer cells [13]. DNA methylation (DNAMe) is a widely accepted gene expression silencing mark and was considered as coupled to H3K27me3 through enzymatic interaction [6]. Genome-wide mapping of DNAMe, however, revealed that most strong CpG island promoters are unmethylated even when they were inactive, and low CpG content promoters are predominantly methylated although this methylation does not preclude gene expression [14]. Furthermore, global profiling of epigenetic silencing marks in prostate cancer cells showed that H3K27me3 modified loci excluded DNA hypermethylation [15,16]. All these recent findings suggest that the complex epigenetic regulation based on histone methylation and DNA methylation is far from understood.

Epigenetic analysis of genes critical for EMT has been performed, but limited to very few genes including E-cadherin. Evidence was first presented that DNA hypermethylation may be a mechanism of E-cadherin inactivation [17]. However, a recent report showed that reversible histone modifications rather than DNA demethylation are the predominant factors in reactivation of E-cadherin expression [18]. Apart from this E-to-N cadherin switch, we also found that P-cadherin, a basal cell-specific epithelial mark [19], is significantly down-regulated in EPT1 cells [4]. Regarding the epigenetic modifications of N-cadherin and P-cadherin genes, very little information is available. Considering the conflicting observations above and that the epigenetic regulation of most of the critical genes of EMT is unknown, genome-wide profiling of the epigenetic modifications during EMT is highly desirable.

Taking advantage of the striking gene expression reprogramming associated with EMT and the subsequent acquisition of defined malignant features of our present model and comprehensive analysis of histone and DNA methylations based on the same promoter microarray platform, we describe here the dynamic epigenetic change patterns of critical genes for EMT and provide the first blueprint of epigenetic modification during EMT in prostate cells.

## Results

### Genome-wide profiling of histone methylation, DNA methylation and gene expression in prostate cells

**Figure 1:**
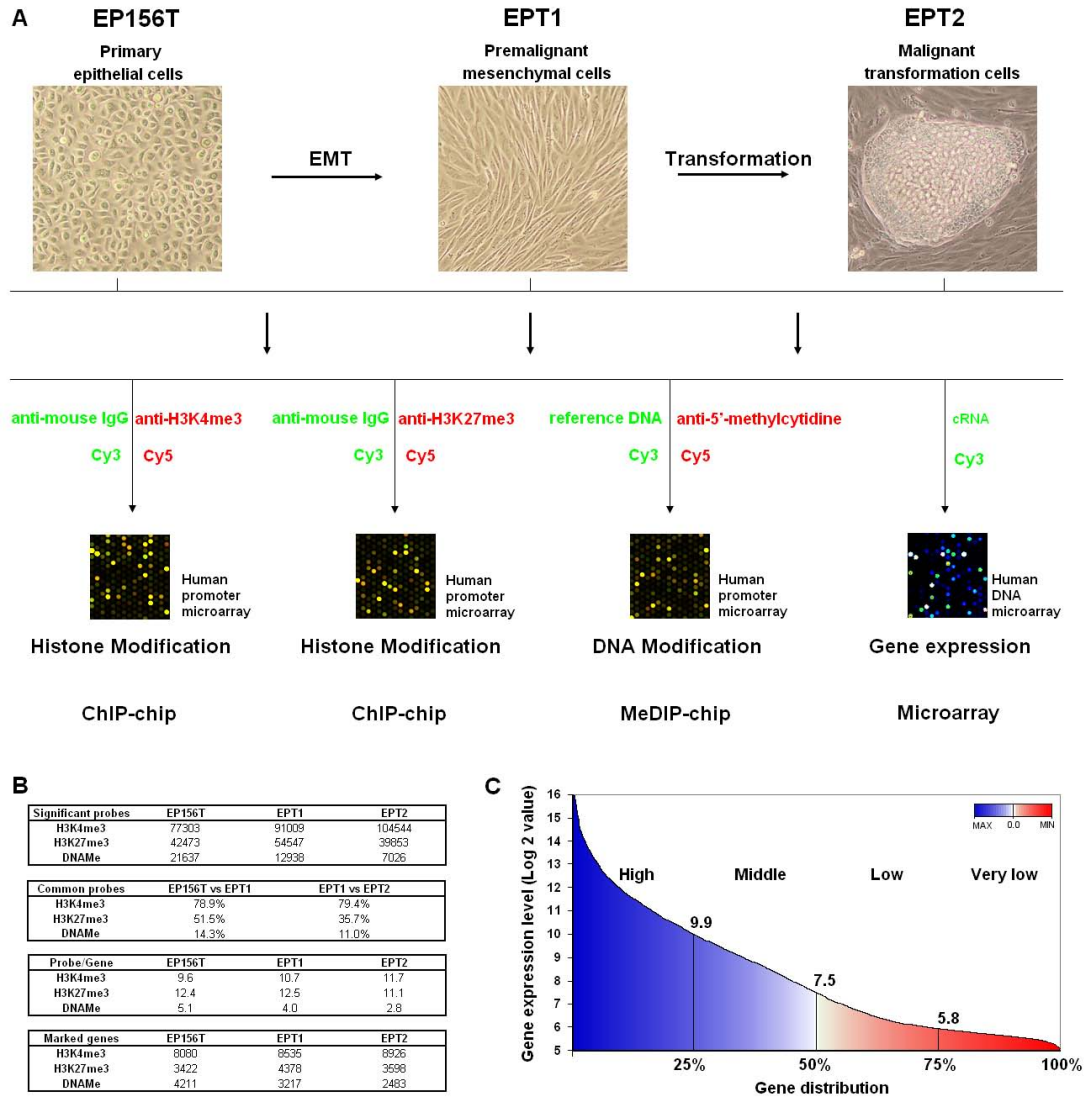
**Overview of genome-wide profiling of histone methylation, DNA methylation and gene expression of prostate cells**. (**A**) Flowchart of ChIP-chip, MeDIP-chip and gene expression microarray profiling of EP156T, EPT1 and EPT2 cells. (**B**) Summary of detected probes and genes that were modified by H3K4me3, H3K27me3 and DNAMe in EP156T, EPT1 and EPT2 cells. Around 20%, 10% and 3% of probes detected significant H3K4me3, H3K27me3 and DNAMe modification, respectively, in the three cell lines. (**C**) Gene distribution according to the expression levels in EP156T cells. Genes with expression level above 7.5 were considered as expressed genes, genes with expression level below 7.5 were considered as silenced genes.

Profiling of histone and DNA methylation of EP156T, EPT1 and EPT2 cells was performed using chromatin immunoprecipitation (ChIP) and methylation DNA immunoprecipitation (MeDIP) protocols followed by human promoter microarrays containing 488 k 60-mer probes, which cover 5.5 kb upstream to 2.5 kb downstream of the transcription start sites of 17,000 defined human RefSeqs (Figure 1A). Histone or DNA modification intensities were indicated by the normalized value of log2 ratios between IP signals and control signals. Probes with intensity above 1 were considered as significant. The quality of human promoter microarray hybridizations was validated by quantitative PCR (Figure S1 in Additional file 1), showing that most of the detected regions have similar modification patterns as found by promoter microarray profiling.

As shown in Figure 1B, near 80% of all significant H3K4me3 probes were in common between EP156T & EPT1 and EPT1 & EPT2 cells, suggesting that H3K4me3 modification patterns were quite similar between these cells, thus underscoring the high reliability of the platform. Much fewer common H3K27me3 and DNAMe probes were detected among EP156T, EPT1 and EPT2 cells, suggesting that these cell lines have very different modification patterns of H3K27me3 and DNAMe. Total intensities including the promoter and gene body were used to define epigenetically marked genes based on a threshold of 7. Calculation of significant probes per marked gene suggested that the modification level of DNA methylation was much lower than that of both H3K4me3 and H3K27me3 in all the three cell lines (Figure 1B).

Global gene expression analysis was achieved using Agilent 44 k human DNA microarrays containing 17596 unique gene symbols. The gene expression levels were indicated by the normalized signal intensities with cutoff 5 for the lowest and 16 for the highest in log2 values (Figure 1C). Previous studies showed that around 25% of genes were highly expressed and 50% of genes were repressed in both normal and cancer human cells [10,16,20]. The median values of gene expression in EP156T, EPT1 and EPT2 cells were quite similar and all were around 7.5 (Additional file 2). So we defined active genes with intensities above 7.5, while silent genes displayed intensities below 7.5.

Comparing the gene lists of the promoter microarrays and gene expression microarrays, 14212 genes were covered in both arrays and were used for further analysis. Approximately 60%, 26% and 22% of all genes were marked by H3K4me3, H3K27me3 and DNAMe, respectively, in all three cell lines (Figure 1B and Additional file 3). To further validate the ChIP-chip and MeDIP-chip data, we selected a set of housekeeping genes [21] and found that these genes are preferably modified by H3K4me3 but not by H3K27me3 and DNA methylation (Table S1 in Additional file 1), which is consistent with previous observations [22].

### DNA Methylation is *per se *only a weak mark of gene repression

**Figure 2:**
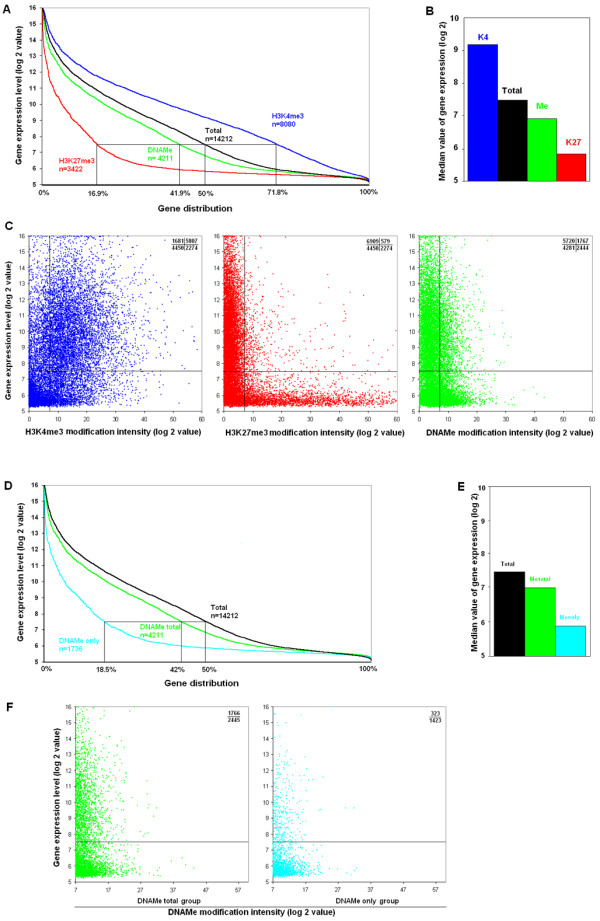
**Correlation between H3K4me3, H3K27me3 and DNAMe modifications and gene activities**. (**A**) Genes were ranked according to their expression levels in EP156T cells. The percentages shown along the × axis correspond to expressed genes for each kind of modification. (**B**) Comparison of gene activities with different epigenetic modifications in EP156T cells. Gene activities were indicated as median values of gene expression levels of each kind of genes. (**C**) Scatter plots between gene expression levels and epigenetic modification levels in EP156T cells. Comparison of the gene distribution (**D**) and the median values of gene expression (**E**) between genes marked by DNAMe without additional H3K4me3 and all genes marked by DNAMe in EP156T cells. (**F**) Scatter plots between DNAMe modification intensities and gene expression levels.

It is established that H3K4me3 is an active mark while both H3K27me3 and DNAMe are marks of silent gene expression [6,23]. We compared the three kinds of epigenetic modifications with gene activities in all three prostate cell lines, as shown for EP156T cells in Figure 2A and 2B. As expected, the expression level of the H3K4me3 marked gene group was much higher than the total level (with median values 9.1 versus 7.5, p < 0.05), and the expression level of the H3K27me3 marked gene group was much lower than the total level (with median values 5.8 versus 7.5, p < 0.05). Surprisingly, the median expression level of the DNA methylated gene group was very close to the total level (6.8 versus 7.5), suggesting that DNA methylation, which was regarded as a silencing epigenetic mark of gene activity, showed only slight correlation with gene repression.

The weak correlation between DNA methylation and gene activities was also confirmed by scatter plot analysis, as exemplified for EP156T cells. A very evident positive and negative correlation was found between H3K4me3 and H3K27me3 and gene expression, respectively (Figure 2C). Up to 78% of expressed genes were modified by H3K4me3, and among H3K4me3 marked genes, 72% of them were expressed. In contrast, 92% of expressed genes were non-H3K27me3 modified, and 80% of H3K27me3 modified genes were silent genes. However, among DNA methylated genes, 42% of genes were expressed and 58% were silenced. Similar relationships were also found in EPT1 and EPT2 cells.

### DNA methylation is a strong silencing mark for genes modified only by DNAMe without H3K4me3

It was very surprising to find that DNA methylation correlated only weakly to gene repression, since DNA methylation is established in silencing of many individual genes [23]. Indeed, we also found that most of the top ten methylated genes were completely silenced in all the three cell lines (see Table S2 in Additional file 1), including RUNX3, whose promoter has been found previously to be DNA hypermethylated in prostate cancer patients [24]. Considering that up to 60% of genes were modified by H3K4me3, we selected genes that were modified only by DNA methylation without the additional H3K4me3 and compared their methylation intensities and gene expression levels. As exemplified for EP156T cells in Figure 2D, among 4211 DNAMe marked genes, 1736 genes were considered DNAMe marked only. The expression levels of these DNAMe only marked genes were significantly lower than the levels of all genes (5.8 versus 7.5 in median values, p < 0.05) (Figure 2E). Scatter plots showed that 81.5% of DNAMe only marked genes were silent genes, which is much higher than the percentage of all DNAMe marked genes (58.0%) (Figure 2F), suggesting that DNA methylation is a strong silencing mark of gene expression when it is not co-modified by the active H3K4me3 mark. Comparable results were found also in EPT1 and EPT2 cells.

### Bivalent H3K4/K27me3 is a repressive mark and H3K4me3/DNAMe is an activation mark in prostate cells

**Figure 3:**
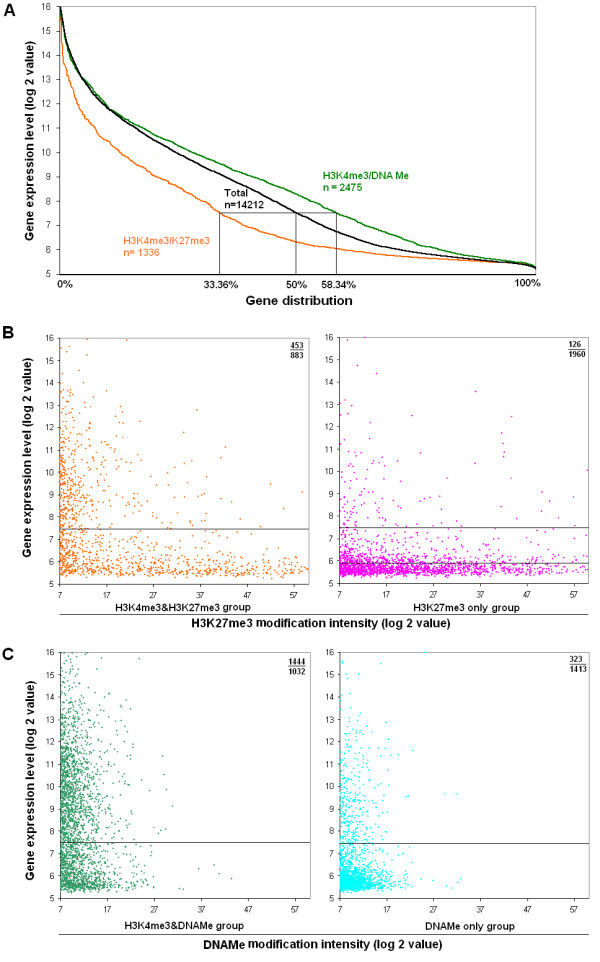
**Correlation between bivalent modifications and gene activities in EP156T cells**. (**A**) Comparison of gene activities of H3K4me3/K27me3 and H3K4me3/DNAMe marked genes and all (Total) genes. (**B**) Comparison of gene activities between H3K4me3/K27me3 bivalently marked genes and H3K27me3 only marked genes using scatter plots. Genes below the lower threshold in the panel to the right belonged to the VL group. (**C**) Comparison of gene activities between bivalent H3K4me3/DNAMe marked genes and DNAMe only marked genes using scatter plots.

Bivalent epigenetic modification was first discovered in embryonic stem cells when genes were modified by both H3K4me3 and H3K27me3 and showed low transcription activity [7-10]. Here, we examined the association between bivalent H3K4/K27me3 as well as H3K4me3/DNAMe and gene expression in somatic cells. As shown in Figure 3A, H3K4/K27me3 modified genes have lower expression levels than all genes and more silenced genes (67%) than expressed genes (33%), suggesting it is a combined silencing mark. This is consistent with previous observations that H3K4/K27me3 bivalent promoters showed low transcription activity. However, genes marked by H3K4me3/DNAMe have expression levels higher than all genes (Figure 3A) and less silenced genes (42%) than expressed genes (58%), suggesting that H3Kme3/DNAMe is a combined activation mark rather than a silencing mark. Scatter plots also showed more silent genes than active genes in the H3K4/K27me3 group (Figure 3B), and more active genes than silent genes in H3K4me3/DNAMe group (Figure 3C). Comparable results were found also in EPT1 and EPT2 cells. It is very interesting to find that genes marked by H3K4me3/DNAMe are preferentially active but not silent genes, which means that misleading conclusions will be drawn if we predict the activities of DNA methylated genes without taking into consideration the H3K4me3 modification.

There are pronounced differences between the H3K27me3 only and H3K4/K27me3 groups although both are silencing marks (Figure 3B). The percentage of silent genes is much higher (94%) among the H3K27me3 only marked genes than among the H3K4/K27me3 bivalent genes (66%, p < 0.05). Furthermore, most of the silenced genes marked by H3K27me3 have very low expression level (< 5.8). It is a reasonable estimate that H3K4/K27me3 marked genes have moderate, but not very low activity in prostate cells. In contrast, most of the DNAMe only genes (81.4%) are silent genes, and more H3K4me3/DNAMe marked genes (58%) are active genes (p < 0.05) (Figure 3C). This finding may explain why DNA methylation correlated only weakly to gene silencing at the global level since most (2475 out of 4211) of the DNAMe genes were co-modified by the active mark H3K4me3 and therefore were more likely to become expressed.

### Transcription activities of genes with bivalent marks depend on the relative intensities of active and repressive marks

Previous work found that H3K4/K27me3 bivalently modified promoters showed low transcription activity and argued that the repressive effect of H3K27me3 is epistatic to the activating effect of H3K4me3 in a bivalent domain [7,8]. However, we have observed that most of the H3K4/K27me3 marked genes have stronger modification signal intensities of H3K27me3 than H3K4me3 and propose that the transcription activities of genes with double marks are reflected by the relative signal intensities of active and repressive marks.

**Figure 4:**
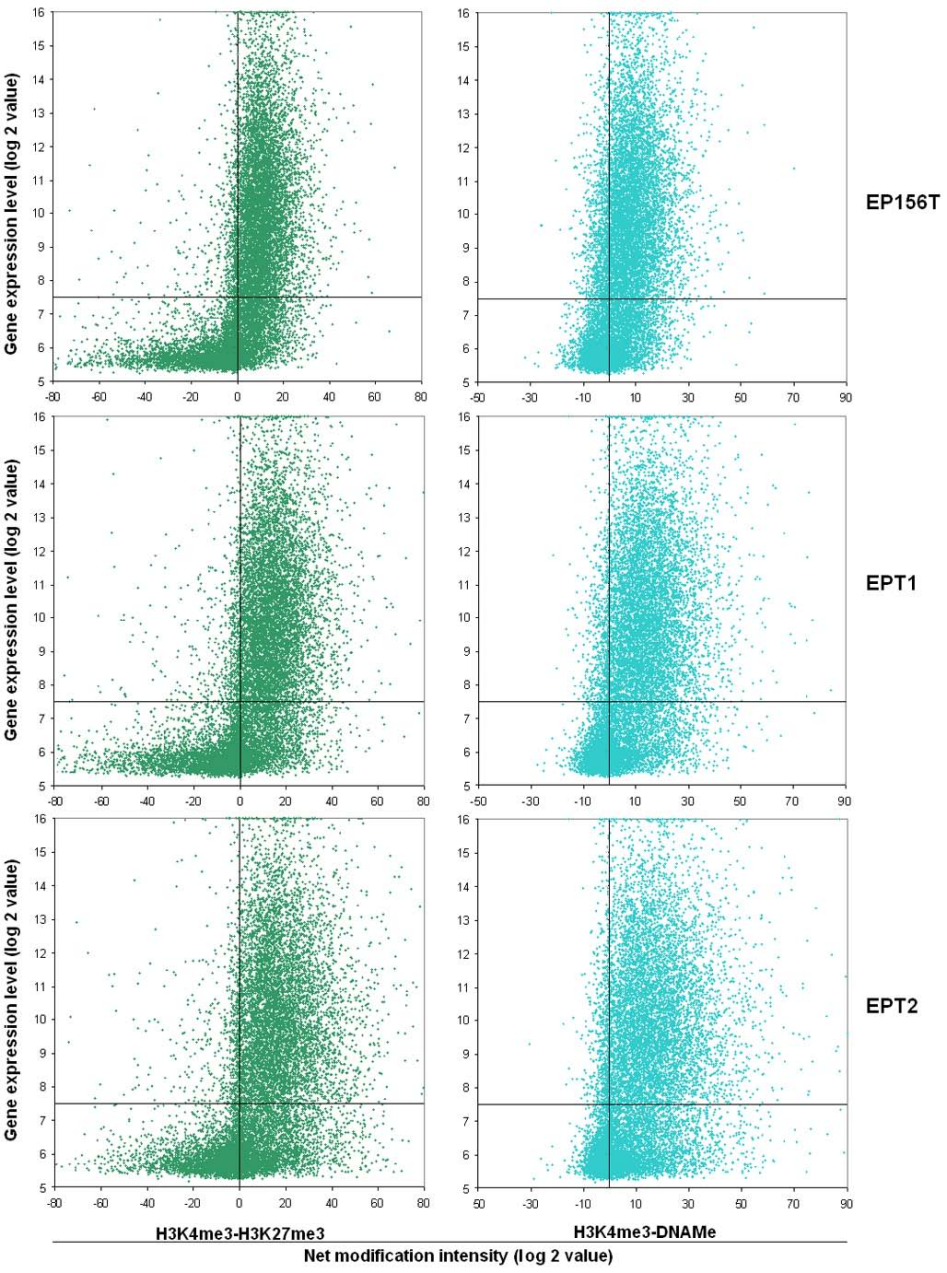
**Correlation between net signal intensity of epigenetic modifications and gene activities of bivalently marked genes**. The net intensities of H3K4me3 versus H3K27me3 or H3K4me3 versus DNAMe are shown along the X-axis. Gene expression levels are shown along the Y-axis. Genes above the horizontal lines are expressed genes. Genes below horizontal lines are considered as repressed genes. Genes to the left of vertical lines are stronger labeled by active marks. Genes to the right of vertical lines are stronger labeled by repressive marks.

To examine this hypothesis, we quantitatively calculated the modification signal intensities of genes with both active and silencing marks. We considered H3K4me3 intensity as the positive value and H3K27me3 or DNAMe intensities as negative values. The net intensity of epigenetic modifications is the sum of positive and negative values. The net epigenetic modification intensities and the gene expression levels of both H3K4/K27me3 and H3K4me3/DNAMe marked genes were plotted for the three cell types (Figure 4). Very strikingly, in the H3K4/K27me3 gene group, as exemplified for EP156T cells, up to 92% of the expressed genes have positive net intensity, which means that most of them have stronger intensity of H3K4me3 than of H3K27me3. When it comes to genes with negative net intensities, meaning that the intensity of H3K27me3 is stronger than of H3K4me3, most (87%) of them are silent genes. Similar patterns were observed for H3K4me3/DNAMe modified genes. Most (86%) of the expressed genes have stronger modification signal intensities of H3K4me3 than DNAMe, and most (79%) of the genes with negative net intensity are silent genes.

The finding that the transcriptional activity of genes with double marks depends of the relative intensities of active and repressive marks suggested that there is not any dominant effect of H3K27me3 over H3K4me3 *per se*. Individual genes are more likely to be active genes if the modification intensities of H3K4me3 are stronger than H3K27me3, implying more modified sites in the same region. In genes with H3K4/H3K27me3 modification, the sum of the H3K27me3 signal intensities (negative values, -25563) is higher than the sum of the H3K4me3 intensities (positive values, 16682), while in genes with H3K4me3/DNAMe, the sum of the DNAMe (negative values, -3885) is lower than the sum of the H3K4me3 signal intensities (positive values, 35335), which may explain, at the global level, why H3K4/H327me3 marked genes showed somewhat lower activity while H3K27me3/DNAMe marked genes showed higher transcription activity.

### H3K4me3 and DNAMe often co-modified genes but at separate regions

Bivalently marked genes as described above have been defined when their promoters contained two different epigenetic marks [7,9]. The fine patterns of co-modification have remained unsettled since one gene might be modified by two marks at two separate regions. Here, we examined the co-localization of double marks with bivalently marked probes that were detected with significant intensities for both marks. Clustered bivalent probes were defined when at least 3 bivalent probes mapped to one gene to distinguish them from more separated sporadic bivalent probes.

**Figure 5:**
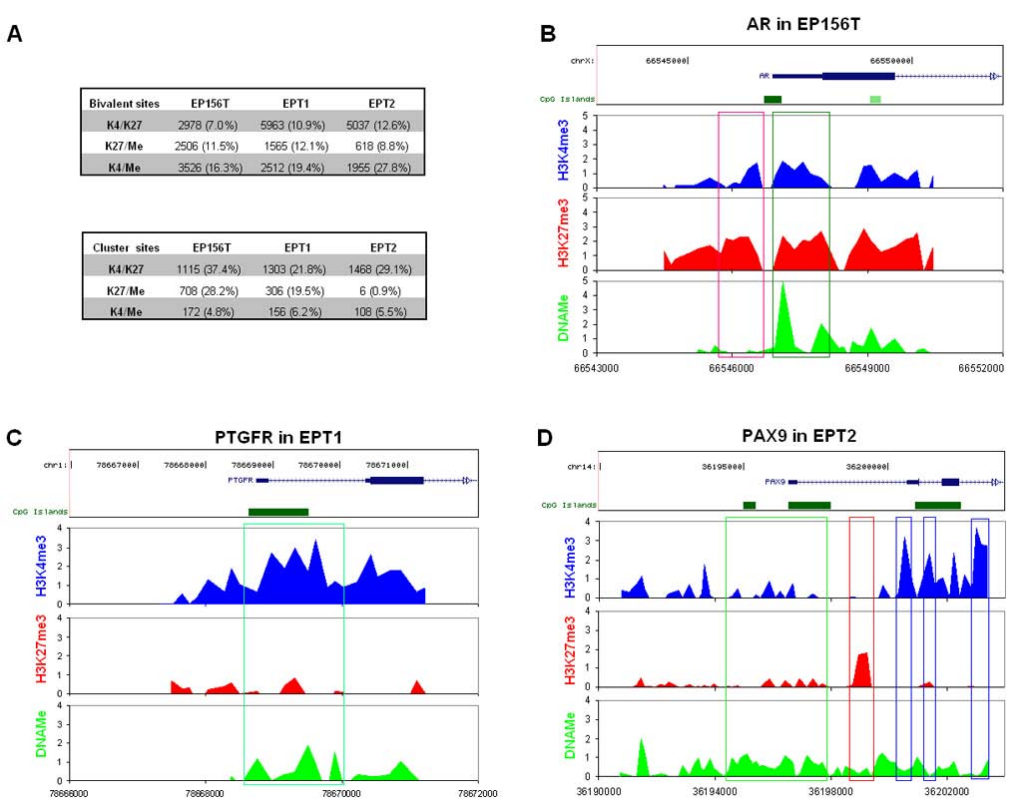
**Examination of bivalently marked sites**. (**A**) Summary of bivalent sites and clustered sites in EP156T, EPT1 and EPT2 cells. (**B**) Epigenetic modifications of the androgen receptor (*AR*) gene in EP156T cells. The red box indicates a bivalent H3K4/K27me3 site. The green box indicates H3K4/K27me3/DNAMe trivalent sites. (**C**) Epigenetic modifications of the prostaglandin F receptor (*PTGFR*) gene in EPT1 cells. The green box shows a bivalent H3K4me3/DNAMe site. (**D**) Epigenetic modifications of the *PAX9 *gene in EPT2 cells. The green box shows DNAMe only modified region, the red box shows an H3K27me3 only modified region and blue boxes show H3K4me3 only modified regions.

As shown in Figure 5A, the bivalent probes with lowest and highest frequency at the global level were H3K4/K27me3 and H3K4me3/DNAMe, respectively. However, bivalent H3K4/K27me3 marks much more often belonged to clustered probes compared to H3K4me3/DNAMe marks, which means that many of the H3K4/K27me3 sites colocalized in certain genes, and most of the H3K4me3/DNAMe sites were distributed separately with limited co-localization. Actually, we found that many of the significant H3K4me3 and H3K27me3 probes cluster together and form big islands, and most of the bivalent H3K4/K27me3 probes were accommodated into these islands (example in Figure 5B), while significant DNAMe probes were often separately distributed with very few clustered DNAMe sites, and the frequency of clustered H3K4me3/DNAMe marks was even lower (Figure 5C). We concluded that in prostate cells, DNAMe labeled probes were more often co-labeled by H3K4me3 than by H3K27me3, but H3K4/K27me3 probes much more frequently clustered within certain genes than H3K4me3/DNAMe probes.

When one gene was modified by both H3K4me3 and DNAMe, such as bivalent genes described in Figure 3 most of the DNAMe marked regions were separated from H3K4me3 islands, with very few bivalent H3K4me3/DNAMe probes (Figure 5D).

### Increased histone methylation and decreased DNA methylation during EMT

**Figure 6:**
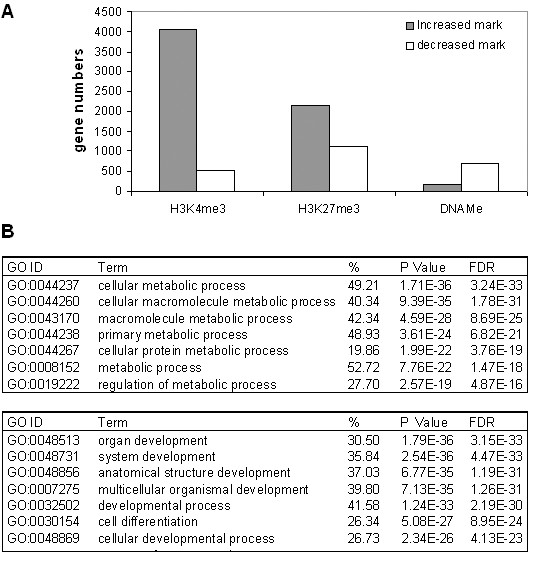
**Analysis of genes with changed epigenetic marks from EP156T to EPT1 cells during EMT**. (**A**) Comparison of numbers of genes with different kind of epigenetic mark changes. (**B**). Gene ontology (GO) analysis of genes with epigenetic mark changes during EMT.

It is interesting to know how epigenetic marks change from EP156T to EPT1 cells during EMT, in which striking cell biological and morphological changes were observed. The changed epigenetic modification was calculated as the difference between the intensity of epigenetic marks in EPT1 cells versus EP156T cells and indicated as delta (Δ). Only genes with Δ values above 5 were considered as differentially epigenetic modified genes. As shown in Figure 6A and Additional file 4, 28.5% of the analyzed genes have increased H3K4me3 marks (4072), while much fewer genes have decreased H3K4me3 marks (534) during EMT. Similar change was also found for the H3K27me3 mark with near twice as many genes with increased marks as genes with decreased marks. In contrast, the DNAMe mark changed in a much lower number of genes, and then more genes exhibited decreased DNAMe marks (706) than increased DNAMe marks (166). The observation of increased H3K4me3 and H3K27me3 modification and decreased DNAMe modification during EMT is also shown in the detected significant probes in EP156T and EPT1 cells (Figure 1B). There were more significant probes of H3K4me3 and H3K27me3 marks and less of DNAMe marks detected in EPT1 cells than in EP156T cells.

Gene ontology was performed to find what kinds of genes have changes of epigenetic modification during EMT. The most enriched GO terms in genes with increased H3K4me3 marks are metabolic process, while the most enriched GO terms in genes with decreased H3K4me3 mark are developmental process (Figure 6B). Development related processes are enriched in genes with both increases and decreases of either H3K27me3 or DNAMe marks (Additional file 4).

### Dynamic changes of H3K4me3 or H3K27me3 but not of DNAMe correlate to changes of gene expression during EMT

**Figure 7:**
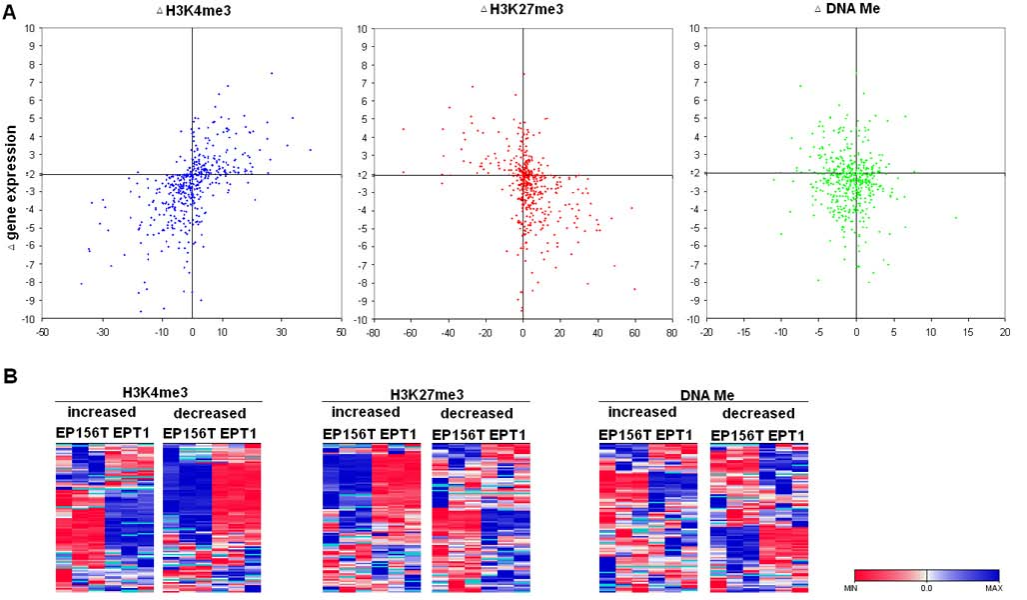
**Correlation between changed epigenetic modifications and changed gene expression during EMT**. (**A**) Scatter plots of the changed epigenetic modification of genes that were differentially expressed between EP156T and EPT1 cells. (**B**) Comparison of the expression patterns of the top 100 epigenetically changed genes during EMT.

To understand epigenetic regulation of EMT at the global level, we examined the dynamic changes of both epigenetic modification and gene expression during EMT. The changed gene expression was also calculated as the Δlog2 value between two cell lines. Only genes with Δ log2 values above 2 were considered as differentially expressed genes. There were 172 up-regulated and 307 down-regulated genes during EMT (Additional file 5). Gene ontology analysis showed that these genes were highly enriched in processes such as ectoderm and epithelium development, cell migration and adhesion (Additional file 5), in which EMT is widely involved [1]. Examining the changed epigenetic modifications among these changed genes, we found that most of the up-regulated genes have increased H3K4me3 and most of the down-regulated genes have decreased H3K4me3 modifications (p < 0.05) (Figure 7A). In contrast, most of these up-regulated genes have decreased H3K27me3 and most down-regulated genes have increased H3K27me3 (p < 0.05). However, there were no significant differences between genes with increased or decreased DNA methylation in either up-regulated or down-regulated genes.

We also examined the changed gene expression of genes with changed epigenetic modifications. The expression levels of the top 100 epigenetically changed genes are shown in Figure 7B. It is very evident that most genes with increased H3K4me3 were up-regulated during EMT, while genes with decreased H3K4me3 were down-regulated during EMT. Opposite changes were found for H3K27me3, in which most of the genes with increased H3K27me3 were down-regulated during EMT, while genes with decreased H3K27me3 were up-regulated during EMT. However, there was no significant correlation between changed DNA methylation and changed gene expression during EMT.

### Epigenetic switches of critical EMT-associated genes

**Figure 8:**
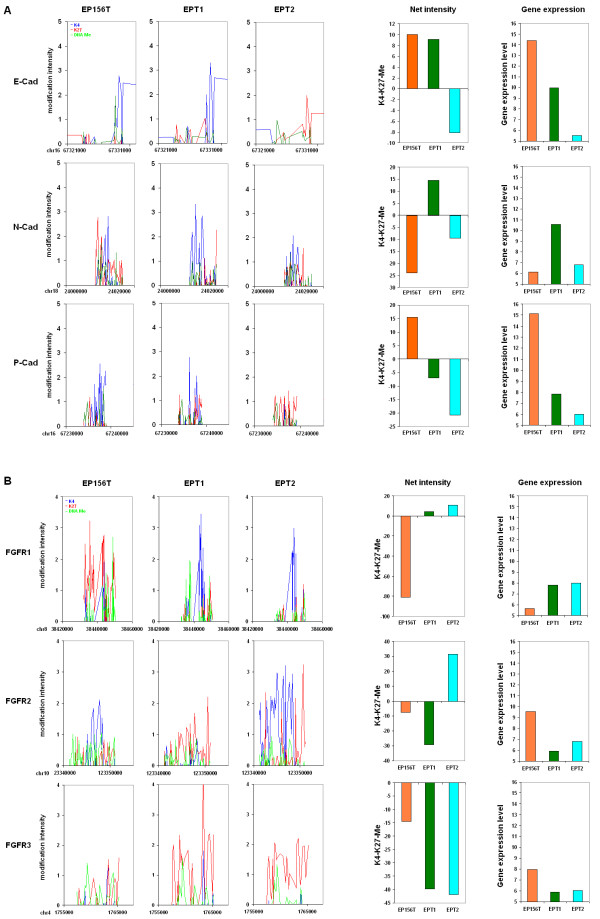
**Epigenetic modification and gene expression of cadherin and *FGFR* families during EMT and early transformation**. (**A**) Epigenetic modification of the promoters (left) and gene expression (right) of E-cadherin (*CDH1*), N-cadherin (*CDH2*) and P-cadherin (*CDH3*) in EP156T, EPT1 and EPT2 cells. (**B**) Epigenetic modification of the promoters (left) and gene expression (right) of *FGFR1*, *FGFR2 *and *FGFR3 *in EP156T, EPT1 and EPT2 cells.

EP156T cells were strikingly reprogrammed during EMT with loss of multiple epithelial markers and gain of mesenchymal features. One of the most characterized switches is the cadherin switch with down-regulation of E-cadherin and gain of N-cadherin. Additionally, we found that P-cadherin, a basal cell-specific epithelial mark [19], is significantly down-regulated in EPT1 cells [4]. Based on the epigenetic profiling, we found that the epigenetic promoter modifications of these three cadherins changed significantly among EP156T, EPT1 and EPT2 cells (Figure 8A). For P-cadherin, its promoter was dominated by H3K4me3 and it was highly expressed in EP156T cells. Following EMT the H3K4me3 modification decreased and the repressive marks H3K27me3 and DNAMe became stronger than H3K4me3 and the expression level of P-cadherin decreased significantly. During the transition from EPT1 to EPT2 cells, H3K4me3 modifications completely disappeared but H3K27me3 increased, and the expression of P-cadherin decreased further. In contrast, the N-cadherin promoter was dominated by repressive marks in EP156T cells, but it became H3K4me3 dominated as the expression level strongly increased in EPT1 cells after EMT. During accumulation of malignant features, H3K4me3 decreased and repressive marks became stronger again. Concomitantly, the expression of N-cadherin reversed to silencing again. For E-cadherin, the epigenetic switch was not significant although the gene expression clearly decreased during EMT. However, the continued decrease of E-cadherin expression from EPT1 to EPT2 cells was accompanied by significant epigenetic switches with complete disappearance of H3K4me3 and increase of H3K27me3. We therefore can report an extensive consistency between epigenetic switches and gene expression switches of N-cadherin and P-cadherin during both EMT and early transformation steps. For E-cadherin this consistency was found only at the early transformation step, but not at the EMT step, suggesting that the E-cadherin promoter is affected by different regulatory subprograms.

Fibroblast growth factor receptor 1 (FGFR1) is a known EMT inducer whose activation can lead to irreversible prostate adenocarcinoma and EMT [25]. Very interestingly, there is also a switch between *FGFR1 *and another *FGFR *member *FGFR3*. The gene expressions between *FGFR1 *and *FGFR3 *were observed as mutually exclusive in colorectal carcinoma cells and indicated that their reciprocal relationship plays an important role in the progression of the carcinomas [26]. *FGFR2 *is also involved in EMT by an alternative splicing event between mutually exclusive exons IIIb and IIIc. The *FGFR2*-IIIb splice variant is exclusive to epithelial cells, while *FGFR2*-IIIc is expressed in mesenchymal cells [27]. In our prostate cell model, we found that the activation of *FGFR1 *was accompanied by significant down-regulation of *FGFR3 *and *FGFR2 *during EMT. This switch was kept from EPT1 to EPT2 cells (Figure 8B). In EP156T cells, the promoter of *FGFR1 *was strongly modified by H3K27me3 and DNAMe, and the transcription was completely silenced, while H3K4me3 modification increased strongly, but DNA methylation decreased and H3K27me3 disappeared during EMT, accompanied by activation of *FGFR1 *expression. In contrast, the promoter of *FGFR2 *underwent the opposite switch with decreased H3K4me3 and increased H3K27me3 during EMT as the expression of *FGFR2*-IIIb was down-regulated. The epigenetic switches in the promoter of *FGFR3 *involved only H3K27me3 and DNA methylation, but not H3K4me3. In EP156T cells, the promoter was modified by H3K27me3 and DNA methylation at very low levels and *FGFR3 *was expressed at middle level, H3K27me3 modification strongly increased during EMT and *FGFR3 *became completely silenced in EPT1 cells. From EPT1 to EPT2 cells, the promoter of *FGFR1 *was continually dominated by H3K4me3 and the expression was still active. The promoter of *FGFR3 *was continually dominated by H3K27me3 and the expression was still repressed.

### Identification of genes with consistent epigenetic switches and expression switches during EMT

Apart from the cadherins and *FGFR*s described above, we also identified multiple other important EMT-associated genes with consistent changes of histone modifications according to their differential expression (Additional file 6). We examined four kinds of combinations including increased H3K4me3 & up-regulated genes, decreased H3K4me3 & down-regulated genes, increased H3K27me3 & down-regulated genes and decreased H3K27me3 & up-regulated genes. Very interestingly, many of these genes have significant changes of both H3K4me3 and H3K27me3 marks. For example, all of the top 9 genes (*LAD1, RBM35A*, *FXYD3, IGSF91, GJB2, EVA1, PCSK9, OVOL2 *and *RNF43*) with increased H3K27me3 marks were accompanied by decreased H3K4me3 modification, and all of the top 5 genes (*FGFR1*, *FOXF2*, *CDH11*, *ATOH8 *and *BDNF*) with decreased H3K27me3 were accompanied by increased H3K4me3 (Additional file 6). In addition, many of them were involved in cell junctions, such as *GJB2*, *4*, *5 *and *6 *of gap junctions, *CDH2*, *3 *and *11 *in cadherin junctions, *DSC3*, *DSG3 PKP1 *and *PKP3 *of desmosomes, as well as *KRT5*, which is expressed especially in the basal layer of the epidermis. All these coordinated epigenetic changes and expression changes of these cell junction genes indicate that histone methylation is very critical in regulating the breakdown of epithelial cell homeostasis and leading to the loss of epithelial characteristics and the acquisition of migratory phenotypes.

## Discussion

This is the first genome-wide study of histone and DNA methylation during EMT with subsequent accumulation of malignant features. EP156T are primary benign prostate epithelial cells, EPT1 are progeny cells that underwent EMT without malignant transformation. EPT2 cells were subsequently derived from EPT1 cells. EPT2 cells acquired several well-defined malignant features, including anchorage independent growth, resistance to apoptosis and independence of external growth factors [5]. The study revealed consistent relationships between promoter modifications and gene expression patterns both at the global level and concerning many individual genes previously known to play important roles in these processes. It therefore appears that epigenetic modifications are involved in the coordination of entire gene expression programs during EMT and early transformation steps. However, EPT2 cells represent only early transformation and did not form tumors in tested animals [5]. The lack of tumorigenicity represents a limitation of this study. Work is ongoing to identify the factors that restrict tumorigenicity in the EPT2 cells even after their acquisition of anchorage independent growth and self sufficiency of growth factors.

Most protein coding genes are modified by at least two of the H3K4me3 and H3K27me3 or DNAMe marks [7-11,14,28,29]. One observation of particular interest is that the net modification signal intensities of activating and repressive marks were quantitatively associated with the modified gene activities. The transcriptional level of genes with bivalent marks depends on the relative intensities of active and repressive marks. This is in contrast to an alternative model of "dominance" of one type of mark over the other [7,8]. Actually, when it comes to genes with stronger H3K4me3 intensity than H3K27me3 intensity, these genes are more likely expressed genes. Hence, it is very important to examine both active and silent marks before correlation of gene expression activities and epigenetic modifications.

It is widely accepted that DNA methylation is a repressive mark of gene activity. However, this concept may have to be modified in several important aspects. For example, DNA methylation at certain upstream or downstream promoter sites was associated with higher expression, which may be due to the inhibition of binding of silencers [30]. Complexity also occurs when DNA methylated sites belong to the intragenic region where it can relate to both gene activity and silencing [31]. We found that DNA methylation is a strong silencing mark only when genes are modified by DNAMe without concomitant H3K4me3, and DNA methylation correlates only slightly to promoter repression at the global level. Furthermore, a gene modified by both DNAMe and H3K4me3 is more likely to be activated. A recent report also found that methylation of DNA and not H3K4 correlated to suppressed transcription, while gene transcription was only slightly reduced when both DNA and H3K4 were methylated [29].

We found that DNA methylation has less significant effect than histone methylation on prostate cells during EMT. Firstly, the number of significant DNAMe-detecting probes was much less than for H3K4me3 and H3K27me3. Secondly, the modification level of DNAMe was much lower than for both H3K4me3 and H3K27me3 in all the three cell lines. Thirdly, more H3K27me3 marked genes (94%) than DNAMe marked genes (81.5%) were repressed, indicating that DNA methylation correlates weaker than H3K27me3 with gene repression. Lastly and most importantly, there was no significant correlation between the changed DNAMe modification and changed gene expression. Actually, the weak correlation between DNA methylation and gene silencing has been observed also when many DNA methylation events in cancer occurred at the promoters of genes that were already repressed in the normal tissue, before transformation [32]. All these findings were different from the prevailing view that DNAMe plays the critical role in silencing gene expression [6,23]. Two technical limitations should be noted, however, since we detected methylated promoter DNA using an antibody against 5'-methylcytidine, which preferably recognizes methylated CpG islands with threshold sensitivity [32]. However, most CpG islands in the genome are not methylated and methylated DNA is more abundantly detected in repeat sequences compared to promoters of protein coding genes [29]. The Agilent promoter array used in our profiling could be associated with loss of some of the CpG islands present in the CpG island array.

More than half of the protein coding genes were modified by both H3K4me3 and DNAMe in our prostate cell lines, Fouse *et al *and Li *et al *also found that 40% and 66%, respectively, of DNAMe marked genes were co-modified by H3K4me3 [28,29], which seems in conflict with an observation that there is a strong anti-correlation between DNA methylation and the presence of H3K4me [6]. This apparent conflict may be explained by our present examination at higher resolution which shows that DNAMe and H3K4me3 marks indeed tend to localize at different sites within the same promoter. This supports that DNAMe and H3K4me3 modifications are really mutually exclusive at the same sites [6], but both marks can colocalize in different regions of the same gene bodies.

Epigenetic regulation of E-cadherin has been addressed for a long time and in many labs with different results. Promoter DNA hypermethylation was the first reported epigenetic silencing mechanism of E-cadherin [17]. Later studies pointed to H3K27me3 rather than DNA demethylation in E-cadherin repression [18,33]. Our findings indicate that epigenetic regulation of the E-cadherin promoter varied at different stages. During EMT in our cell culture model, none of the H3K4me3, H3K27me3 or DNAMe marks were changed in the promoter of E-cadherin even though the transcription of E-cadherin was significantly down-regulated. During subsequent accumulation of malignant features, we detected complete loss of H3K4me3 accompanied with full silencing of E-cadherin expression.

## Conclusions

EMT is a crucial event in tumor progression. This work presents the first blueprint of epigenetic modifications during EMT in prostate cells and shows that specific histone methylations are extensively involved in gene expression reprogramming during EMT and subsequent accumulation of malignant features. Many genes, especially epithelial cell junction genes, showed corresponding changes concerning epigenetic modifications and gene expression during EMT. The observation that transcription activity of bivalently marked genes depends on the relative labeling intensity of each mark provides a new view of quantitative regulation of epigenetic modification.

## Methods

### Cell lines and cell culture

The prostate cell lines EP156T, EPT1 and EPT2 were grown in modified MCDB153 medium as described [4,5].

### Chromatin immunoprecipitation (ChIP)

ChIP was performed according to the Agilent ChIP-chip protocol with modifications as previously described [13]. To immunoprecipitate chromatin, 6 × 10^7 ^cells were treated with 1% formaldehyde at room temperature for 10 min followed by quenching with 0.125 M glycine. Cells were lysed and the nuclei were sonicated under conditions yielding DNA fragments ranging from 200 to 800 basepairs. Five percent of the sonicated material was saved as whole cell extract. Sonicated lysate was divided into three equal volumes and immunoprecipitated with specific or non-specific antibody bound to protein A magnetic beads (Invitrogen) overnight at 4°C with rocking. Antibodies used were against: H3K4me3 (Abcam no. ab8580) or H3K27me3 (Abcam no. ab6002) or mouse IgG (Sigma). Five μg of antibody was used per 2 × 10^7^cells. Immunoprecipitated complexes were collected, washed and eluted using the Dynal Magnetic Particle Concentrator (Invitrogen). Eluted DNA and whole-cell extracts were incubated at 65°C in a rotating incubator for 8 hours to reverse cross-links. DNA samples were sequentially treated with RNase A and proteinase K and then purified by phenol/chloroform extraction. The immunoprecipitated (ChIPed) and purified DNA was ethanol precipitated using glycogen as a carrier and resuspended in nucleic acid grade water.

### Methylated DNA immunoprecipitation (MeDIP)

MeDIP was performed according to the Agilent Microarray Analysis of Methylated DNA Immunoprecipitation protocol with modifications. Briefly, genomic DNA was purified using SDS/Proteinase K incubation, phenol:chloroform extraction, ribonuclease A incubation and phenol:chloroform extraction followed by ethanol precipitation. Purified genomic DNA was sonicated to 200-600 bp in size. Thirty μg sonicated DNA was incubated with 200 μl pan-mouse IgG Dynal magnetic beads (Invitrogen) coupled to 20 *μ*g antibody against 5'-methylcytosine (Eurogentec, Seraing, Belgium) overnight at 4°C. The genomic DNA-beads-antibody complex was washed and methylated DNA was eluted using the Dynal Magnetic Particle Concentrator. Eluted DNA and reference DNA were extracted using phenol:chloroform:isoamylalcohol and precipitated with ethanol.

### Human promoter microarray profiling

For each microarray, 2 μg ChIPed DNA or MeDIPed DNA and reference DNA were labeled with Cy3-dUTP or Cy5-dUTP (GE Healthcare) using the CGH kit (Invitrogen). Human G4489A 2 × 244 k promoter arrays (Agilent) were hybridized for 40 h at 65°C (for ChIP) or 67°C (for MeDIP) and subsequently scanned using an Agilent Scanner controlled by Agilent Scan Control 7.0 software. Raw image files were extracted with Agilent Feature Extraction 9.1 software.

### Real-time quantitative PCR

Real-time quantitative PCR was performed to validate the ChIP-chip and MeDIP-chip data using TaqMan assays (Applied Biosystems, Foster City USA). The relative fold enrichment of target regions was calculated based on the differences in Ct values (ΔCt) between ChIPed or MeDIPed DNA and reference DNA that were pulled down by mouse IgG. Fold change = 2^ΔCt^. Three biological and technical replicates were done for each sample. All values were expressed as mean ± standard deviation.

### Global gene expression analysis

The Agilent Human Whole Genome (4 × 44 k) Oligo Microarray with Sure Print Technology (Agilent Technologies, Palo Alto USA) was used to analyze samples in the present study as previously described [4]. Quality and yields of total RNA were assessed using the Agilent 2100 Bioanalyzer (Agilent Tech), 1% agarose gel ethidium bromide electrophoresis and Powerwave spectrophotometry at 260 nm and 280 nm. One μg of DNAse-treated total RNA was converted into cDNA and Cy3-labeled cRNA using the Low RNA Input Linear Amplification Kit PLUS, One-Color kit (Agilent Tech.) according to instructions. We used gmeansignals, *i.e*. signals without background subtraction. Intraarray normalization of dye effects was carried out using quantile normalization [34] and genes with more than 25% missing values were removed. The normalized signal values were log2 transformed with cutoff 5 for the lowest level and 16 for the highest level in gene expression. Data were formatted in a J-Express-file suitable for additional data mining (http://www.molmine.com/).

### Gene ontology (GO) analysis

GO Analysis was performed as described previously [5,13].

### Data analysis

ChIP-chip and MeDIP-chip data were analyzed using Agilent's ChIP Analytics 1.3 software. Peaks were detected using Whitehead Per-array Neighborhood Model v1.0. Maximum distance for two probes to be considered as neighbors is 1000 bp, a probe is considered "bound" if P(Xbar) < 0.001 and central probe has P(X) < 0.001 and at least one neighboring probe has P(X) < 0.1. To achieve quantitative analysis of epigenetic modifications, total signal intensities of epigenetic marks were calculated as follows: 1) All negative intensity values were considered as zero. 2) All intensity values including promoter and gene body of each gene were summarized. 3) Genes with total intensities above 7 were defined as marked genes. Mapping of bound probes was performed using human genome HG17, May 2004.

### Statistical analysis

Pearson's Chi-square test was used in the analysis of the correlation between epigenetic modification and gene expression in prostate cells. The significance was defined by a p-value ≤ 0.05. Differences were considered statistically significant for *p *< 0.05. All calculations were carried out using the SPSS software.

### Accession numbers

Gene Expression study ArrayExpress accession number: E-TABM-949; ChIP-chip study ArrayExpress accession numbers: E-TABM-635; E-TABM-983; MeDIP-chip study ArrayExpress accession number: E-TABM-982.

## Abbreviations

EMT: epithelial to mesenchymal transition; H3K4me3: histone H3 lysine 4 tri-methylation; H3K27me3: histone H3 lysine 27 tri-methylation; DNAMe: DNA methylation; ChIP: Chromatin immunoprecipitation; MeDIP: methylation DNA immunoprecipitation.

## Authors' contributions

XSK, YQ and KHK designed the research. XSK YC AMO and KHK performed experiments, collected and analyzed data. XSK, YQ and KHK wrote the manuscript. All authors read and approved the final manuscript.

## Authors' details

XSK is PhD and research fellow funded by the Bergen Medical Research Foundation; YQ is PhD student funded by the University of Bergen; YC is bioinformatician in Heidelberg, Germany; WCL is PhD and urological surgeon in Wuhan, China; VR is PhD, Professor and Head of the Department of Molecular Cell Biology in Rehovot, Israel; AMO is PhD and research fellow funded by Helse Vest, Haukeland University Hospital, Bergen; KHK is MD&PhD, Professor and Senior consultant in virology at Haukeland University Hospital, Bergen, Norway.

## Supplementary Material

Additional file 1 **Validation of ChIP-chip and MeDIP-chip data**. This file contains 1 figure of Chip-qPCR and 2 tables. Click here for file

Additional file 2 **Gene expression of EP156T, EPT1 and EPT2 cells**. The expression levels are indicated by the normalized signal intensities with cutoff 5 for the lowest and 16 for the highest in log2 values. Click here for file

Additional file 3 **Epigenetic modifications of EP156T, EPT1 and EPT2 cells**. The intensities of epigenetic modification (H3K4me3, H3K27me3 and DNAMe) are shown as the log2 ratio between IP samples and reference samples. Click here for file

Additional file 4 **Epigenetic changed genes during EMT and gene ontology analysis**. The file contains the list of genes with epigenetic changes including both increased and decreased H3K4me3, H3K27me3 and DNAMe from EP156T to EPT1 cells. Click here for file

Additional file 5 **Expression changed genes during EMT and geneontology analysis**. This file contains the list of genes that have changed expression from EP156T to EPT1 cells and the GO analysis of these genes. Click here for file

Additional file 6 **Gene list of the top epigenetic changed genes with consistent changes in gene expression level during EMT**. This list shows genes that are most likely regulated by epigenetic modification from EP156T cells to EPT1 cells. Click here for file

## References

[B1] Thiery JP Epithelial-mesenchymal transitions in tumour progression Nat Rev Cancer 2002 2 6 442 454 10.1038/nrc822 12189386

[B2] Thiery JP Sleeman JP Complex networks orchestrate epithelial-mesenchymal transitions Nat Rev Mol Cell Biol 2006 7 2 131 142 10.1038/nrm1835 16493418

[B3] Yang J Weinberg RA Epithelial-mesenchymal transition: at the crossroads of development and tumor metastasis Dev Cell 2008 14 6 818 829 10.1016/j.devcel.2008.05.009 18539112

[B4] Ke XS Qu Y Goldfinger N Rostad K Hovland R Akslen LA Rotter V Oyan AM Kalland KH Epithelial to Mesenchymal Transition of a Primary Prostate Cell Line with Switches of Cell Adhesion Modules but without Malignant Transformation PloS ONE 2008 3 10 1 11 10.1371/journal.pone.0003368 PMC2557125 18852876

[B5] Ke XS Li WC Hovland R Qu Y Liu RH McCormack E Thorsen F Olsen JR Molven A Kogan-Sakin I Reprogramming of cell junction modules during stepwise epithelial to mesenchymal transition and accumulation of malignant features in vitro in a prostate cell model Exp Cell Res 2011 317 234 247 10.1016/j.yexcr.2010.10.009 20969863

[B6] Cedar H Bergman Y Linking DNA methylation and histone modification: patterns and paradigms Nat Rev Genet 2009 10 5 295 304 10.1038/nrg2540 19308066

[B7] Bernstein BE Mikkelsen TS Xie X Kamal M Huebert DJ Cuff J Fry B Meissner A Wernig M Plath K A bivalent chromatin structure marks key developmental genes in embryonic stem cells Cell 2006 125 2 315 326 10.1016/j.cell.2006.02.041 16630819

[B8] Mikkelsen TS Ku M Jaffe DB Issac B Lieberman E Giannoukos G Alvarez P Brockman W Kim TK Koche RP Genome-wide maps of chromatin state in pluripotent and lineage-committed cells Nature 2007 448 7153 553 560 10.1038/nature06008 17603471 PMC2921165

[B9] Pan G Tian S Nie J Yang C Ruotti V Wei H Jonsdottir GA Stewart R Thomson JA Whole-genome analysis of histone H3 lysine 4 and lysine 27 methylation in human embryonic stem cells Cell Stem Cell 2007 1 3 299 312 10.1016/j.stem.2007.08.003 18371364

[B10] Zhao XD Han X Chew JL Liu J Chiu KP Choo A Orlov YL Sung WK Shahab A Kuznetsov VA Whole-genome mapping of histone H3 Lys4 and 27 trimethylations reveals distinct genomic compartments in human embryonic stem cells Cell Stem Cell 2007 1 3 286 298 10.1016/j.stem.2007.08.004 18371363

[B11] Wei G Wei L Zhu J Zang C Hu-Li J Yao Z Cui K Kanno Y Roh TY Watford WT Global mapping of H3K4me3 and H3K27me3 reveals specificity and plasticity in lineage fate determination of differentiating CD4+ T cells Immunity 2009 30 1 155 167 10.1016/j.immuni.2008.12.009 19144320 PMC2722509

[B12] Cui K Zang C Roh TY Schones DE Childs RW Peng W Zhao K Chromatin signatures in multipotent human hematopoietic stem cells indicate the fate of bivalent genes during differentiation Cell Stem Cell 2009 4 1 80 93 10.1016/j.stem.2008.11.011 19128795 PMC2785912

[B13] Ke XS Qu Y Rostad K Li WC Lin B Halvorsen OJ Haukaas SA Jonassen I Petersen K Goldfinger N Genome-wide profiling of histone h3 lysine 4 and lysine 27 trimethylation reveals an epigenetic signature in prostate carcinogenesis PLoS ONE 2009 4 3 e4687 10.1371/journal.pone.0004687 19262738 PMC2650415

[B14] Weber M Hellmann I Stadler MB Ramos L Paabo S Rebhan M Schubeler D Distribution, silencing potential and evolutionary impact of promoter DNA methylation in the human genome Nat Genet 2007 39 4 457 466 10.1038/ng1990 17334365

[B15] Gal-Yam EN Egger G Iniguez L Holster H Einarsson S Zhang X Lin JC Liang G Jones PA Tanay A Frequent switching of Polycomb repressive marks and DNA hypermethylation in the PC3 prostate cancer cell line Proc Natl Acad Sci USA 2008 105 35 12979 12984 10.1073/pnas.0806437105 18753622 PMC2529074

[B16] Komashko VM Acevedo LG Squazzo SL Iyengar SS Rabinovich A O'Geen H Green R Farnham PJ Using ChIP-chip technology to reveal common principles of transcriptional repression in normal and cancer cells Genome Res 2008 18 4 521 532 10.1101/gr.074609.107 18347325 PMC2279240

[B17] Yoshiura K Kanai Y Ochiai A Shimoyama Y Sugimura T Hirohashi S Silencing of the E-cadherin invasion-suppressor gene by CpG methylation in human carcinomas Proc Natl Acad Sci USA 1995 92 16 7416 7419 10.1073/pnas.92.16.7416 7543680 PMC41350

[B18] Yang X Pursell B Lu S Chang TK Mercurio AM Regulation of beta 4-integrin expression by epigenetic modifications in the mammary gland and during the epithelial-to-mesenchymal transition J Cell Sci 2009 122 Pt 14 2473 2480 10.1242/jcs.049148 19549682 PMC2704882

[B19] Jarrard DF Paul R van Bokhoven A Nguyen SH Bova GS Wheelock MJ Johnson KR Schalken J Bussemakers M Isaacs WB P-Cadherin is a basal cell-specific epithelial marker that is not expressed in prostate cancer Clin Cancer Res 1997 3 11 2121 2128 9815605

[B20] Su AI Wiltshire T Batalov S Lapp H Ching KA Block D Zhang J Soden R Hayakawa M Kreiman G A gene atlas of the mouse and human protein-encoding transcriptomes Proc Natl Acad Sci USA 2004 101 16 6062 6067 10.1073/pnas.0400782101 15075390 PMC395923

[B21] Eisenberg E Levanon EY Human housekeeping genes are compact Trends Genet 2003 19 7 362 365 10.1016/S0168-9525(03)00140-9 12850439

[B22] She X Rohl CA Castle JC Kulkarni AV Johnson JM Chen R Definition, conservation and epigenetics of housekeeping and tissue-enriched genes BMC Genomics 2009 10 269 10.1186/1471-2164-10-269 19534766 PMC2706266

[B23] Esteller M Cancer epigenomics: DNA methylomes and histone-modification maps Nat Rev Genet 2007 8 4 286 298 10.1038/nrg2005 17339880

[B24] Richiardi L Fiano V Vizzini L De Marco L Delsedime L Akre O Tos AG Merletti F Promoter methylation in APC, RUNX3, and GSTP1 and mortality in prostate cancer patients J Clin Oncol 2009 27 19 3161 3168 10.1200/JCO.2008.18.2485 19470943

[B25] Acevedo VD Gangula RD Freeman KW Li R Zhang Y Wang F Ayala GE Peterson LE Ittmann M Spencer DM Inducible FGFR-1 activation leads to irreversible prostate adenocarcinoma and an epithelial-to-mesenchymal transition Cancer Cell 2007 12 6 559 571 10.1016/j.ccr.2007.11.004 18068632

[B26] Jang JH Reciprocal relationship in gene expression between FGFR1 and FGFR3: implication for tumorigenesis Oncogene 2005 24 5 945 948 10.1038/sj.onc.1208254 15558020

[B27] Warzecha CC Sato TK Nabet B Hogenesch JB Carstens RP ESRP1 and ESRP2 are epithelial cell-type-specific regulators of FGFR2 splicing Mol Cell 2009 33 5 591 601 10.1016/j.molcel.2009.01.025 19285943 PMC2702247

[B28] Fouse SD Shen Y Pellegrini M Cole S Meissner A Van Neste L Jaenisch R Fan G Promoter CpG methylation contributes to ES cell gene regulation in parallel with Oct4/Nanog, PcG complex, and histone H3 K4/K27 trimethylation Cell Stem Cell 2008 2 2 160 169 10.1016/j.stem.2007.12.011 18371437 PMC3070208

[B29] Li X Wang X He K Ma Y Su N He H Stolc V Tongprasit W Jin W Jiang J High-resolution mapping of epigenetic modifications of the rice genome uncovers interplay between DNA methylation, histone methylation, and gene expression Plant Cell 2008 20 2 259 276 10.1105/tpc.107.056879 18263775 PMC2276441

[B30] Bloushtain-Qimron N Yao J Snyder EL Shipitsin M Campbell LL Mani SA Hu M Chen H Ustyansky V Antosiewicz JE Cell type-specific DNA methylation patterns in the human breast Proc Natl Acad Sci USA 2008 105 37 14076 14081 10.1073/pnas.0805206105 18780791 PMC2532972

[B31] Shann YJ Cheng C Chiao CH Chen DT Li PH Hsu MT Genome-wide mapping and characterization of hypomethylated sites in human tissues and breast cancer cell lines Genome Res 2008 18 5 791 801 10.1101/gr.070961.107 18256232 PMC2336806

[B32] Keshet I Schlesinger Y Farkash S Rand E Hecht M Segal E Pikarski E Young RA Niveleau A Cedar H Evidence for an instructive mechanism of de novo methylation in cancer cells Nat Genet 2006 38 2 149 153 10.1038/ng1719 16444255

[B33] Cao Q Yu J Dhanasekaran SM Kim JH Mani RS Tomlins SA Mehra R Laxman B Cao X Kleer CG Repression of E-cadherin by the polycomb group protein EZH2 in cancer Oncogene 2008 27 58 7274 7284 10.1038/onc.2008.333 18806826 PMC2690514

[B34] Bolstad BM Irizarry RA Astrand M Speed TP A comparison of normalization methods for high density oligonucleotide array data based on variance and bias Bioinformatics 2003 19 2 185 193 10.1093/bioinformatics/19.2.185 12538238

