# Supplementary material for: Global profiling of histone and DNA methylation reveals epigenetic-based regulation of gene expression during epithelial to mesenchymal transition in prostate cells
Source: BMC Genomics. 2010 Nov 25;11:669. doi: 10.1186/1471-2164-11-669 (PMC3012672; doi:10.1186/1471-2164-11-669)
Supplement: Additional file 1 — Validation of ChIP-chip and MeDIP-chip data. This file contains 1 figure of Chip-qPCR and 2 tables. [file 1471-2164-11-669-S1.DOC]

**
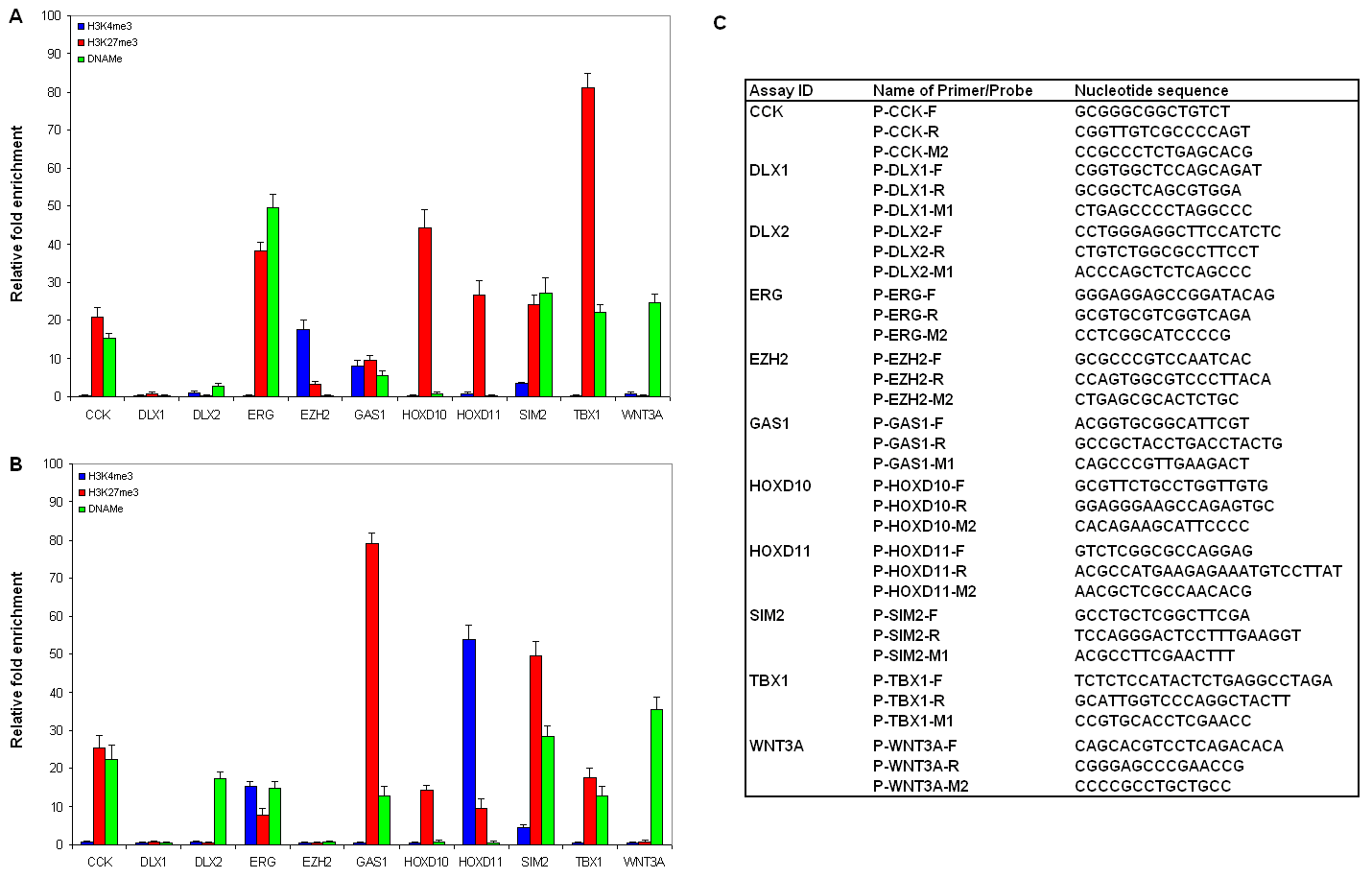
Figure S1. Validation of ChIP or MeDIP samples by quantitative real-time PCR (qPCR).** H3K4me3, H3K27me3 and DNAMe modification of 11 randomly selected genes were detected by qPCR in EPT1 (**A**) and EPT2 (**B**) cells, respectively. The qPCR results of these genes in EP156T cells were described previously [12]. Relative fold enrichment was calculated based on the ΔCt values between the immunoprecipitated DNA and reference DNA (see Materials and methods). All the results represent three independent triplicate experiments as mean ± standard deviation. (**C**) The nucleotide sequences of Taqman probes used in qPCR. F means the forward primer, R means the reverse primer, M means minor groove binder (MGB) probe.

**Table S1. Comparison of the percentages of epigenetic marked genes between**

**housekeeping (HK) genes and all genes in EP156T, EPT1 and EPT2 cells**

| **Cell line** | **Modification** | **All genes** | **HK genes** |
| --- | --- | --- | --- |
| **EP156T** | **H3K4me3** | 56.85% | 76.91% |
|  | **H3K27m3** | 24.08% | 6.50% |
|  | **DNAMe** | 29.63% | 17.26% |
| **EPT1** | **H3K4me3** | 60.05% | 82.51% |
|  | **H3K27m3** | 30.80% | 15.92% |
|  | **DNAMe** | 22.64% | 11.88% |
| **EPT2** | **H3K4me3** | 62.80% | 81.17% |
|  | **H3K27m3** | 25.32% | 9.87% |
|  | **DNAMe** | 17.47% | 7.62% |

**Table S2. The gene expression of the top 10 DNA methylated genes in EP156T, EPT1 and EPT2 cells**

| **EP156T** | | | **EPT1** | | | **EPT2** | | |
| --- | --- | --- | --- | --- | --- | --- | --- | --- |
| **Gene** | **DNAMe** | **Expression** | **Gene** | **DNAMe** | **Expression** | **Gene** | **DNAMe** | **Expression** |
| DMD | 43.24 | 5.89 | DMD | 49.96 | 6.10 | DMD | 56.51 | 5.84 |
| CREB5 | 40.74 | 6.09 | PLEC1 | 39.42 | 6.05 | ARSA | 49.79 | 9.29 |
| DTNA | 39.63 | 6.50 | ARPP-21 | 34.80 | 5.64 | FOXP2 | 47.77 | 5.48 |
| PLEC1 | 36.30 | 6.34 | NDP | 33.35 | 5.49 | FBXW7 | 39.09 | 6.52 |
| ARPP-21 | 32.95 | 5.40 | RGS12 | 31.15 | 6.19 | RUNX1T1 | 35.60 | 5.44 |
| BGN | 32.66 | 5.87 | RUNX3 | 30.90 | 5.43 | PAX9 | 34.08 | 6.54 |
| FOXP2 | 32.29 | 5.42 | BGN | 30.34 | 6.38 | SOX6 | 33.75 | 5.29 |
| TNS1 | 31.98 | 5.73 | TNS1 | 30.32 | 5.60 | NDP | 33.00 | 5.46 |
| RGS12 | 31.84 | 6.19 | TSC22D3 | 30.08 | 8.90 | LMO3 | 31.85 | 5.70 |
| RUNX3 | 30.93 | 5.44 | SORBS3 | 28.86 | 11.54 | FGF13 | 31.54 | 6.33 |
